# Relationship between circadian syndrome and stroke: A cross-sectional study of the national health and nutrition examination survey

**DOI:** 10.3389/fneur.2022.946172

**Published:** 2022-08-11

**Authors:** Yuling Wang, Ling Yang, Yan Zhang, Junyan Liu

**Affiliations:** Department of Neurology, Third Hospital of Hebei Medical University, Shijiazhuang, China

**Keywords:** circadian syndrome, stroke, correlation, (NHANES) database, symptom

## Abstract

**Aim:**

The aim of this study was to assess the relationship of circadian syndrome and stroke.

**Methods:**

We performed a cross-sectional analysis of 11,855 participants from the National Health and Nutrition Examination Survey (NHANES) database between 2005 and 2018, and collected the baseline characteristics. Multivariate logistic regression models were developed to explore the association between circadian syndrome and stroke. Simultaneously, subgroup analyses based on the difference of gender, race, and components associated with circadian syndrome also were performed. The odds ratio (OR) and 95% CI were calculated in this study.

**Results:**

All the participants were divided into the non-stroke group and the stroke group. There were approximately 3.48% patients exclusively with stroke and 19.03% patients exclusively with circadian syndrome in our study. The results suggested that the risk of stroke in patients with circadian syndrome was higher than that in patients without circadian syndrome (OR = 1.322, 95 CI%: 1.020–1.713). Similar associations were found in women with circadian syndrome (OR = 1.515, 95 CI%: 1.086–2.114), non-Hispanic whites with circadian syndrome (OR = 1.544, 95 CI%: 1.124–2.122), participants with circadian syndrome who had elevated waist circumference (OR = 1.395, 95 CI%: 1.070–1.819) or short sleep (OR = 1.763, 95 CI%: 1.033–3.009).

**Conclusion:**

Circadian syndrome was associated with the risk of stroke. Particularly, we should pay more close attention to the risk of stroke in those populations who were female, non-Hispanic whites, had the symptoms of elevated waist circumference or short sleep.

## Introduction

Stroke is the leading cause of mortality and serious long-term disability worldwide and has been considered one of the most prevalent and devastating diseases affecting humanity today (1, 2). According to the report of the Global Burden of Disease, the number of patients who were diagnosed with stroke has continued to increase in recent years, resulting in a significant economic burden (3). Existing evidence suggested the risk factors related to stroke, such as arterial hypertension, dyslipidemia, diabetes mellitus, obesity, sleep disorders (4, 5). A better understanding of the contribution of risk factors to stroke burden is important for effective prevention strategies.

The circadian system plays an important role in human health and metabolism (6). Some poor lifestyles, including sleep disturbances, the use of artificial light, and shift work, have been reported to cause the circadian rhythm disturbances, which have adverse effects on human health (7). Recently, a growing number of studies have focused on the relationship between the circadian system and chronic diseases (8–10). Circadian dysfunction has been proposed and defined as the presence of any four of the following seven traits, including elevated waist circumference, elevated triglycerides, reduced high-density lipoprotein (HDL)-cholesterol, elevated blood pressure, elevated fasting glucose, short sleep duration (<6 h/day), and the depression symptom (7). Shi et al. (11) assessed the association between circadian syndrome and cardiovascular disease (CVD), and the result also suggested that the circadian syndrome was a strong predictor for CVD occurrence. However, to the best of our knowledge, there has been no study that examined the association between circadian syndrome and stroke so far.

Herein, the aim of this study was to explore the correlation between circadian syndrome and the occurrence of stroke, and focusing on the number of symptoms in circadian syndrome. In addition, we performed subgroup analyses based on gender, race, and components associated with circadian syndrome.

## Methods

### Data sources and study design

All data in this study were derived from the National Health and Nutrition Examination Survey (NHANES) database between 2005 and 2018. NHANES, as a major program of the National Center for Health Statistics (NCHS), aims to evaluate the health and nutrition of adults and children in the United States (12, 13). The survey adopts a complex multistage sampling method every year to extract nationally representative data of ~5,000 people (14). The data collection process in NHANES contained two parts, an in-person interview (including demographic, socioeconomic, dietary, and health-related questions) and a physical examination (including medical, dental, and physiological measurements, as well as laboratory tests) performed in the Mobile Examination Center (MEC) (14, 15). https://www.cdc.gov/nchs/nhanes/about_nhanes.htm.

Our cross-sectional study included 12,826 participants whose surveys included information on stroke history and characteristics of circadian syndrome. Meanwhile, the exclusion criteria were as follows: (1) subjects without the information of height, weight, marital status, the educational level, household income, smoking, and sleep disorders (*n* = 518); (2) the participants had missing information on heart failure, coronary heart disease (CHD), hypertension, and diabetes mellitus (*n* = 453). All the participants provided written consent to participate in the NHANES survey, and data collection was approved by the NCHS Research Ethics Review Committee.

### Data collection

We collected the following information in the current study: age (years), gender, body mass index (BMI, kg/m^2^), race, marital status, the educational level, household income, smoking, sleep disorders, type of dietary intake [dietary fiber, fat, protein, fruit, vegetables, vitamin A (mcg), vitamin C (mg), vitamin D (mg), vitamin E (mg)], the history of diseases [heart failure, CHD, angina, heart disease, hypertension, high cholesterol, diabetes mellitus], biomarkers [total cholesterol (TC, mg/dL), glycosylated hemoglobin (HbA1c, %), LDL (mg/dL), C-reactive protein (CRP, mg/dL)], and circadian syndrome. Sleep duration was assessed by the question, “How much sleep do you usually get at night on weekdays or workdays?” (16). Sleep disorders were identified by the question “Have you ever been told by a doctor or other health professional that you have a sleep disorder?”. The participants who answered “yes” were asked to say the type of sleep disorder (sleep apnea, insomnia, restless legs, and others) (17, 18). Hypertension was defined as systolic blood pressure of ≥140 mmHg and/or diastolic blood pressure of ≥90 mmHg or the using of antihypertensive medications (19).

#### Definition of circadian syndrome

Having ≥ 4 of the following components was defined as having circadian syndrome (11): elevated waist circumference (≥102 cm in men, ≥88 cm in women), elevated triglycerides (≥150 mg/dL) or the using of lipid-lowering medication, reduced high-density lipoprotein (HDL)-cholesterol (<40 mg/dL in men and <50 mg/dL in women) or the using of lipid-lowering medication, elevated blood pressure (systolic ≥ 130 and/or diastolic ≥85 mmHg) or the using of an anti-hypertensive drug, elevated fasting glucose (≥100 mg/dL) or the using of anti-diabetic medication, short sleep (<6 h/day), depression symptoms [the patient health status questionnaire-9 (PHQ-9) score ≥ 10] (20, 21).

To estimate the association between the symptoms of circadian syndrome and stroke, we defined the circadian syndrome exposure numbers based on the number of components each participant experienced (elevated waist circumference, elevated triglycerides, reduced HDL cholesterol, elevated blood pressure, elevated fasting glucose, short sleep, and depression symptoms): 4 indicated that an exposure of circadian syndrome was composed of four components; 5 indicated circadian syndrome consists of five components; ≥6 represented circadian syndrome had more than six factors. Noteworthily, <4 means that the participants were not defined as circadian syndrome.

#### Outcome

The primary outcome of our study was the occurrence of stroke. Stroke was defined as through the question: “Has a doctor or other health professional ever told you that you had a stroke?”. The participant was considered as a stroke victim when the response was “yes” of the question (5, 22).

### Statistical analysis

After weighted analysis, all measurement data were approximately normal distribution. The measurement data were shown by mean ± standard error (Mean ± SE), and the independent-samples t-test was used for the comparison between two groups. The enumeration data were described as the number of cases and the composition ratio [*n* (%)], comparison between groups adopted χ^2^ or the Fisher's exact test. Additionally, we also interpolated the missing value by multiple Imputation (R: mice), and the sensitivity analysis after interpolation is shown in Supplementary Table 1.

First of all, we performed the univariate difference analysis in the study. Then, variables with statistical significance in univariate difference analysis were included as covariables in the multivariate logistic regression model to explore the correlation between circadian syndrome and stroke, and assess the association between the number of symptoms of circadian syndrome and stroke. Three models were introduced: Model 1 was the coarse model; Model 2 adjusted age and BMI; Model 3 adjusted age, BMI, race, marital status, the educational level, household income, sleep disorders, heart failure, CHD, angina, heart disease, hypertension, high cholesterol, diabetes mellitus, smoking, the intake of dietary fiber, the intake of protein, the intake of fat, the intake of vitamin D, the intake of vitamin E, the intake of fruits, TC, HbA1c, and LDL. Simultaneously, we also conducted subgroup analyses based on the difference of gender, race, and components associated with circadian syndrome. The odds ratio (OR) and 95% confidence interval (CI) were calculated. SAS (version 9.4) software was used for statistical analyses. All statistical tests were conducted by using bilateral tests. *p* < 0.05 was considered as statistically significant difference.

## Results

### Baseline characteristics

After excluding some participants who had the missing information (*n* = 971), a total of 11,855 eligible participants were enrolled eventually, and they were divided into the non-stroke group (*n* = 11,443) and the stroke group (*n* = 412). The incidence of stroke approximately was 3.48% in our study. Baseline characteristics of all the subjects were displayed in Table 1. Overall, the average age was 47.45 ± 0.29 years. Approximately, 2,473 participants were identified as having circadian syndrome, of which 1,614 participants with circadian syndrome were identified by four features, and 711 participants were identified by five features. Moreover, Table 1 also reveals that some variables had statistical significance between the non-stroke group and the stroke group (*p* < 0.05).

**Table 1:** Baseline characteristics of all participants.

**Variables**	**Total** **(*n* = 11,855)**	**Groups**		**Statistics**	***P***
		**Stroke group** **(*n* = 412)**	**Non-stroke group** **(*n* = 11,443)**		
Age, years, Mean ± SE	47.45 ± 0.29	63.9 ± 0.93	46.99 ± 0.28	*t* = −12.380	<0.001
Gender				χ^2^=3.019	0.086
Men	5,857 (49.51)	193 (46.84)	5,664 (49.50)		
Women	5,998 (50.49)	219 (53.16)	5,779 (50.50)		
BMI, kg/m^2^, *n* (%)				χ^2^ = 5.894	0.125
<18.5	180 (1.51)	5 (1.21)	175 (1.53)		
18.5–	3,265 (28.16)	97 (23.54)	3,168 (27.69)		
25–	3,970 (33.56)	119 (28.88)	3,851 (33.65)		
≥30	4,440 (36.77)	191 (46.36)	4,249 (37.13)		
Race, *n* (%)				χ^2^ = 40.471	<0.001
Mexican American	1,814 (8.56)	35 (8.50)	1,779 (15.55)		
Other race	2,569 (12.87)	49 (11.89)	2,520 (22.02)		
Non-Hispanic white	5,142 (68.60)	207 (50.24)	4,935 (43.13)		
Non-Hispanic black	2,330 (9.98)	121 (29.37)	2,209 (19.30)		
Marital status, *n* (%)				χ^2^ = 55.780	<0.001
Married	6,149 (56.05)	216 (52.43)	5,933 (51.85)		
Widowed/divorced	2,532 (17.56)	147 (35.68)	2,385 (20.84)		
Unmarried	2,164 (17.89)	32 (7.77)	2,132 (18.63)		
Cohabitation	1,010 (8.50)	17 (4.13)	993 (8.68)		
Educational level, *n* (%)				χ^2^ = 29.465	<0.001
Junior high and below	2,766 (15.27)	140 (33.98)	2,626 (22.95)		
High school/GED	2,678 (22.63)	105 (25.49)	2,573 (22.49)		
Graduate and above	6,411 (62.10)	167 (40.53)	6,244 (54.57)		
Household income, *n* (%)				χ^2^ = 12.705	0.001
<20,000$	2,794 (16.35)	135 (32.77)	2,659 (23.24)		
≥20,000$	9,061 (83.65)	277 (67.23)	8,784 (76.76)		
Smoking, *n* (%)	5,294 (44.70)	253 (61.41)	5,041 (44.05)	χ^2^ = 26.210	<0.001
Sleep disorders, *n* (%)	1,702 (16.08)	99 (24.03)	1,603 (14.01)	χ^2^ = 21.855	<0.001
Heart failure, *n* (%)	336 (2.19)	71 (17.23)	265 (2.32)	χ^2^ = 36.789	<0.001
CHD, *n* (%)	473 (3.38)	69 (16.75)	404 (3.53)	χ^2^ = 28.363	<0.001
Angina, *n* (%)	273 (2.01)	43 (10.44)	230 (2.01)	χ^2^ = 19.093	<0.001
Heart disease, *n* (%)	475 (3.17)	84 (20.39)	391 (3.42)	χ^2^ = 27.854	<0.001
Hypertension, *n* (%)	4,239 (32.21)	305 (74.03)	3,934 (34.38)	χ^2^ = 70.054	<0.001
High cholesterol, *n* (%)	4,206 (34.38)	247 (59.95)	3,959 (34.60)	χ^2^ = 42.532	<0.001
Diabetes mellitus, *n* (%)	1,574 (9.89)	134 (32.52)	1,440 (12.58)	χ^2^ = 44.722	<0.001
Type of dietary intake, Mean ± SE					
Dietary fiber	17.06 ± 0.19	14.41 ± 0.49	17.13 ± 0.19	t = 5.340	<0.001
Fat	85.51 ± 0.59	74.49 ± 3.39	85.81 ± 0.59	t = 3.410	0.001
Protein	84.19 ± 0.54	68.92 ± 2.42	84.61 ± 0.53	t = 6.570	<0.001
Fruit	1.58 ± 0.02	1.41 ± 0.07	1.58 ± 0.02	t = 2.400	0.018
Vegetable	0.90 ± 0.02	0.82 ± 0.07	0.91 ± 0.02	t = 1.170	0.247
Vitamin A	605.44 ± 7.25	575.46 ± 30.94	606.26 ± 7.24	t = 1.010	0.313
Vitamin C	75.93 ± 1.15	69.35 ± 5.13	76.12 ± 1.15	t = 1.290	0.199
Vitamin E	8.77 ± 0.10	7.31 ± 0.36	8.81 ± 0.10	t = 4.160	<0.001
Vitamin D	4.46 ± 0.07	3.87 ± 0.23	4.48 ± 0.07	t = 2.630	0.010
Biomarkers, Mean ± SE					
TC	192.47 ± 0.65	184.5 ± 2.68	192.69 ± 0.67	t = 2.820	0.006
GHb	5.63 ± 0.01	6.11 ± 0.09	5.62 ± 0.01	t = −5.580	<0.001
LDL	113.55 ± 0.47	104.56 ± 2.33	113.8 ± 0.48	t = 3.750	<0.001
CRP	1.90 ± 0.06	2.38 ± 0.34	1.89 ± 0.06	t = −1.460	0.149
Circadian syndrome, *n* (%)				χ^2^ = 23.680	<0.001
No	9,382 (80.97)	271 (65.78)	9,111 (79.62)		
Yes	2,473 (19.03)	141 (34.22)	2,332 (20.38)		
Components of circadian syndrome				Z = 7.179	<0.001
<4	9,382 (79.14)	271 (65.78)	9,111 (79.62)		
4	1,614 (13.61)	79 (19.17)	1,535 (13.41)		
5	711 (6.00)	41 (9.95)	670 (5.86)		
≥6	133 (1.12)	17 (4.13)	116 (1.01)		

BMI, body mass index; GED, General Equivalent Diploma; CHD, coronary heart disease; TC, total cholesterol; GHb, glycosylated hemoglobin; LDL, low-density lipoprotein; CRP, C-reactive protein.

### The association of circadian syndrome and stroke

As illustrated in Table 2, the results suggested that the risk of stroke in the patients with circadian syndrome was higher than that in the patients without circadian syndrome (Model 1: OR = 2.050, 95 CI%: 1.587–2.648). After adjusting some covariates, the results of Model 2 (OR = 1.539, 95 CI%: 1.186–1.996) and Model 3 (OR = 1.322, 95 CI%: 1.020–1.713) were similar to those of Model 1. In addition, we also found when the patients with circadian syndrome composed of more than six components, the risk of stroke was 2.591-times higher than the patients with circadian syndrome composed of four components (OR = 3.591, 95 CI%: 1.972–6.538).

**Table 2:** The influence of circadian syndrome on stroke.

**Variables**	**Model 1**		**Model 2**		**Model 3**	
	**OR (95%CI)**	***P***	**OR (95%CI)**	***P***	**OR (95%CI)**	***P***
Circadian syndrome						
No						
Yes	2.050 (1.587–2.648)	<0.001	1.539 (1.186–1.996)	0.001	1.322 (1.020–1.713)	0.033
Components of circadian syndrome						
4	Ref		Ref		Ref	
5	1.282 (0.770–2.133)	0.336	1.287 (0.757–2.189)	0.358	1.318 (0.757–2.294)	0.325
≥6	3.365 (1.883–6.015)	<0.001	3.916 (2.123–7.223)	<0.001	3.591 (1.972–6.538)	<0.001

OR, odds ratio; CI, confidence interval.

Model 1: coarse model.

Model 2: adjusted age and body mass index (BMI).

Model 3: adjusted age, BMI, race, marital status, the educational level, household income, sleep disorders, heart failure, coronary heart disease (CHD), angina, heart disease, hypertension, high cholesterol, diabetes mellitus, smoking, the intake of dietary fiber, the intake of protein, the intake of fat, the intake of vitamin D, the intake of vitamin E, the intake of fruit, total cholesterol (TC), glycosylated hemoglobin (GHb), and low-density lipoprotein (LDL).

### The association of circadian syndrome and stroke based on gender, race, and components associated with circadian syndrome

**Figure 1:**
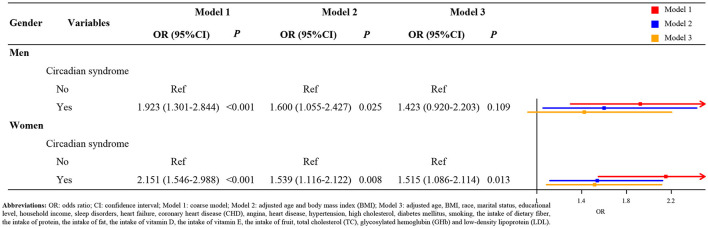
The association of circadian syndrome and stroke based on gender.

**Figure 2:**
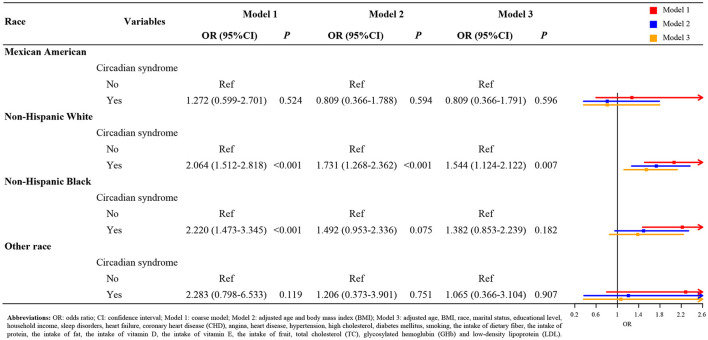
The association of circadian syndrome and stroke based on the race.

**Figure 3:**
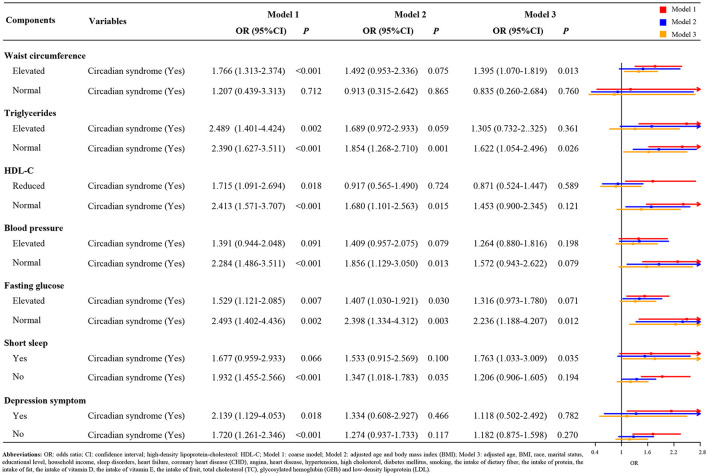
The association of circadian syndrome and stroke based on the components associated with circadian syndrome.

Figure 1 indicates the result of subgroup analysis based on the gender. After adjusting covariates, we found that the risk of stroke in the female patients with circadian syndrome was 1.515 times higher than the patients without circadian syndrome (Model 3: OR = 1.515, 95 CI%: 1.086–2.114, *p* < 0.05). Nevertheless, there was not a statistical significance between male patients with circadian syndrome and stroke (Model 3, *p* > 0.05). Similarly, from the results of the racial subgroup analysis (Figure 2), the patients with circadian syndrome had a higher risk of stroke than those without circadian syndrome only for non-Hispanic whites (Model 3: OR = 1.544, 95 CI%: 1.124–2.122, *p* < 0.05). It is worth noting that we also investigated the correlation between stroke and components associated with circadian syndrome. As shown in Figure 3, the results illustrated that the patients with circadian syndrome with the characteristic of elevated waist circumference had higher risk of stroke compared to the normal waist circumference (Model 3: OR = 1.395, 95 CI%: 1.070–1.819, *p* < 0.05); the patients with circadian syndrome with the characteristic of having short sleep were associated with the higher risk of stroke than those without short sleep (Model 3: OR = 1.763, 95 CI%: 1.033–3.009, *p* < 0.05).

## Discussion

Stroke is recognized as one of the most devastating diseases affecting human civilization, which produced a high effect on the health and quality of life for humans (23). At present, the concept of circadian syndrome has been proposed, which might bring some risk for diseases. However, there were few studies to investigate the association of circadian syndrome and the risk of stroke to date. In this cross-sectional study, the findings showed that circadian syndrome might be associated with the incidence of stroke, especially for female patients with circadian syndrome, non-Hispanic white patients with circadian syndrome, patients with circadian syndrome, who had the characteristic of elevated waist circumference or having short sleep.

To the best of our knowledge, this is the first study conducted to explore the association of circadian syndrome and stroke. Previous studies have only focused on the impact of metabolic syndrome on the risk of stroke (24, 25). However, compared to metabolic syndrome, the circadian syndrome included two components of short sleep and depression symptom. Several reports have illustrated that short sleep and depression symptom could increase the risk of stroke (26, 27). As expected, circadian syndrome was related to the risk of stroke in our study. Because circadian syndrome was defined as meeting at least four characteristics, we discussed the association between the number of characteristics and stroke risk. Interestingly, our study results displayed who patients with circadian syndrome composed of more than six components had a higher risk of stroke compared to the patients with circadian syndrome composed of four components (OR = 3.591, 95 CI%: 1.972–6.538). Although we obtained no significant correlation between patients with circadian syndrome composed of five components and the prevalence of stroke (*p* > 0.05), the OR suggested that stroke risk in patients with circadian syndrome composed of five components may be higher than the circadian syndrome of four components, lower than the circadian syndrome of six components. This suggests that there might be a positive correlation between the number of components each participant with circadian syndrome and stroke experienced. In other words, the greater the number of matching characteristics in patients with circadian syndrome, the higher the risk of stroke may be. However, more prospective studies are still needed to confirm this positive association.

Additionally, the gender and race differences in the association between circadian syndrome and the risk of stroke were observed in this study. The result indicated that the association was more significant in female patients with circadian syndrome. This may be related to gender differences in arterial structure and function (28). In a stratified analysis of the seven features composed the circadian syndrome, we found that not all factors were associated with the risk of stroke; we guessed that the reason may be related to our sample size. It is important to note that patients with circadian syndrome who had the characteristic of elevated waist circumference or short sleep seem to have higher risk on the occurrence of stroke. These findings indicated that future stroke prevention for patients with circadian syndrome may benefit from improving the sleep duration and shrinking the waist circumference, such as increase physical activity, adhere to a regular schedule, reduce the intake of high-calorie foods, and reduce sedentary time.

This study has some advantages. Firstly, our study included a relatively large sample size, which was sufficient to support our conclusion. Also, gender, race, and components were selected for subgroup analysis to provide a more detailed analysis of the impact of different populations diagnosed with circadian syndrome on the risk of stroke. However, our study has some limitations. Firstly, since this was a cross-sectional study, the causal relationship between circadian syndrome and the risk of stroke cannot be determined in this study. Secondly, we excluded some participants who had the missing information; we are not sure whether these missing cases affected the result of this study. Thirdly, the assessment of sleep duration and stroke relied on the participants' self-reported, which might cause an information bias. Lastly, although our study has a relatively large sample size, it does not support the conclusion which factors constitute a stronger association between circadian syndrome and the risk of stroke. Thus, future studies should investigate the causal relationship between circadian syndrome and the risk of stroke in a large-scale population-based cohort study.

## Conclusion

In short, circadian syndrome was associated with the risk of stroke. Of note, we should pay more close attention to the risk of stroke in those populations who are female patients with circadian syndrome, non-Hispanic white patients with circadian syndrome, and patients with circadian syndrome who had the characteristic of elevated waist circumference or having short sleep.

## Data availability statement

Publicly available datasets were analyzed in this study. This data can be found here: https://www.cdc.gov/nchs/nhanes/index.htm.

## Ethics statement

Ethical review and approval was not required for the study on human participants in accordance with the local legislation and institutional requirements. Written informed consent from the patients/participants or patients/participants legal guardian/next of kin was not required to participate in this study in accordance with the national legislation and the institutional requirements.

## Author contributions

YW and JL: designed the study. YW: wrote the manuscript. LY and YZ: collected, analyzed, and interpreted the data. JL: critically reviewed, edited, and approved the manuscript. All authors read and approved the final manuscript.

## Funding

This research was supported by the Hebei Provincial Health Commission (No. 20150716).

## Conflict of interest

The authors declare that the research was conducted in the absence of any commercial or financial relationships that could be construed as a potential conflict of interest.

## Publisher's note

All claims expressed in this article are solely those of the authors and do not necessarily represent those of their affiliated organizations, or those of the publisher, the editors and the reviewers. Any product that may be evaluated in this article, or claim that may be made by its manufacturer, is not guaranteed or endorsed by the publisher.

## References

[1] Iadecola C Buckwalter MS Anrather J. Immune responses to stroke: mechanisms, modulation, and therapeutic potential. J Clin Invest. (2020) 130:2777–88. 10.1172/jci135530 32391806 PMC7260029

[2] Titova OE Michaëlsson K Larsson SC. Sleep Duration and stroke: prospective cohort study and mendelian randomization analysis. Stroke. (2020) 51:3279–85. 10.1161/strokeaha.120.029902 32895015 PMC7587241

[3] Roth GA Mensah GA Johnson CO Addolorato G Ammirati E Baddour LM. Global burden of cardiovascular diseases and risk factors, 1990–2019: update from the GBD 2019 study. J Am Coll Cardiol. (2020) 76:2982–3021. 10.1016/j.jacc.2020.11.010 33309175 PMC7755038

[4] Li J Li L Lv Y Kang Y Zhu M Wang W. Effect of the interaction between depression and sleep disorders on the stroke occurrence: an analysis based on national health and nutritional examination survey. Behav Neurol. (2021) 2021:6333618. 10.1155/2021/6333618 34712368 PMC8548119

[5] MaiXLiangX. Risk factors for stroke based on the national health and nutrition examination survey. J Nutr Health Aging. (2020) 24:791–5. 10.1007/s12603-020-1430-432744577

[6] Thosar SS Butler MP Shea SA. Role of the circadian system in cardiovascular disease. J Clin Invest. (2018) 128:2157–67. 10.1172/JCI80590 29856365 PMC5983320

[7] Zimmet P Alberti K Stern N Bilu C El-Osta A Einat H. The Circadian Syndrome: is the Metabolic Syndrome and much more! J Intern Med. (2019) 286:181–91. 10.1111/joim.12924 31081577 PMC6851668

[8] Fodor DM Marta MM Perju-Dumbravă L. Implications of circadian rhythm in stroke occurrence: certainties and possibilities. Brain Sci. (2021) 11:865. 10.3390/brainsci11070865 34209758 PMC8301898

[9] Walker WH Walton JC DeVries AC Nelson RJ. Circadian rhythm disruption and mental health. Transl Psychiatry. (2020) 10:28. 10.1038/s41398-020-0694-0 32066704 PMC7026420

[10] Videnovic A Zee PC. Consequences of circadian disruption on neurologic health. Sleep Med Clin. (2015) 10:469–80. 10.1016/j.jsmc.2015.08.004 26568123 PMC4648713

[11] Shi Z Tuomilehto J Kronfeld-Schor N Alberti GK Stern N El-Osta A. The circadian syndrome predicts cardiovascular disease better than metabolic syndrome in Chinese adults. J Intern Med. (2021) 289:851–60. 10.1111/joim.13204 33340184

[12] Tucker LA. Physical activity and telomere length in U.S. men and women: an NHANES investigation. Prev Med. (2017) 100:145–51. 10.1016/j.ypmed.2017.04.027 28450121

[13] Taylor MK Mahnken JD Sullivan DK. NHANES 2011-2014 reveals cognition of US older adults may benefit from better adaptation to the mediterranean diet. Nutrients. (2020) 12:1929. 10.3390/nu12071929 32610616 PMC7399952

[14] Cai S Fan J Zhu L Ye J Rao X Fan C. Bone mineral density and osteoporosis in relation to all-cause and cause-specific mortality in NHANES: a population-based cohort study. Bone. (2020) 141:115597. 10.1016/j.bone.2020.115597 32814125

[15] Wu CC Wang CK Yang AM Lu CS Lin CY. Selenium status is independently related to bone mineral density, FRAX score, and bone fracture history: NHANES, 2013 to 2014. Bone. (2021) 143:115631. 10.1016/j.bone.2020.115631 32920174

[16] Chaudhary NS Grandner MA Jackson NJ Chakravorty S. Caffeine consumption, insomnia, and sleep duration: results from a nationally representative sample. Nutrition. (2016) 32:1193–9. 10.1016/j.nut.2016.04.005 27377580 PMC6230475

[17] Huyett P Siegel N Bhattacharyya N. Prevalence of sleep disorders and association with mortality: results from the NHANES 2009–2010. Laryngoscope. (2021) 131:686–9. 10.1002/lary.28900 32681735

[18] Bansil P Kuklina EV Merritt RK Yoon PW. Associations between sleep disorders, sleep duration, quality of sleep, and hypertension: results from the National Health and Nutrition Examination Survey, 2005 to 2008. J Clin Hypertens (Greenwich). (2011) 13:739–43. 10.1111/j.1751-7176.2011.00500.x 21974761 PMC8108844

[19] O'Shea PM Griffin TP Fitzgibbon M. Hypertension: The role of biochemistry in the diagnosis and management. Clin Chim Acta. (2017) 465:131–43. 10.1016/j.cca.2016.12.014 28007614

[20] Iranpour S Sabour S. Inverse association between caffeine intake and depressive symptoms in US adults: data from National Health and Nutrition Examination Survey (NHANES) 2005–2006. Psychiatry Res. (2019) 271:732–9. 10.1016/j.psychres.2018.11.004 30791349

[21] Li W Taskin T Gautam P Gamber M Sun W. Is there an association among sleep duration, nap, and stroke? Findings from the China Health and Retirement Longitudinal Study. Sleep Breath. (2021) 25:315–23. 10.1007/s11325-020-02118-w 32562171

[22] Wang L Li S Sanika GHA Zhao J Zhang H Zhao L. Association between serum 25-hydroxyvitamin D level and stroke risk: an analysis based on the National Health and Nutrition Examination Survey. Behav Neurol. (2021) 2021:5457881. 10.1155/2021/5457881 34745384 PMC8570893

[23] Aigner A Grittner U Rolfs A Norrving B Siegerink B Busch MA. Contribution of established stroke risk factors to the burden of stroke in young adults. Stroke. (2017) 48:1744–51. 10.1161/strokeaha.117.016599 28619986

[24] Li X Li X Lin H Fu X Lin W Li M. Metabolic syndrome and stroke: a meta-analysis of prospective cohort studies. J Clin Neurosci. (2017) 40:34–8. 10.1016/j.jocn.2017.01.018 28268148

[25] Zhang F Liu L Zhang C Ji S Mei Z Li T. Association of metabolic syndrome and its components with risk of stroke recurrence and mortality: a meta-analysis. Neurology. (2021) 97:e695–705. 10.1212/wnl.0000000000012415 34321360

[26] He Q Sun H Wu X Zhang P Dai H Ai C. Sleep duration and risk of stroke: a dose-response meta-analysis of prospective cohort studies. Sleep Med. (2017) 32:66–74. 10.1016/j.sleep.2016.12.012 28366344

[27] Gill D James NE Monori G Lorentzen E Fernandez-Cadenas I Lemmens R. Genetically determined risk of depression and functional outcome after ischemic stroke. Stroke. (2019) 50:2219–22. 10.1161/strokeaha.119.026089 31238828

[28] Asayama K Kikuya M Schutte R Thijs L Hosaka M Satoh M. Home blood pressure variability as cardiovascular risk factor in the population of Ohasama. Hypertension. (2013) 61:61–9. 10.1161/hypertensionaha.111.00138 23172933 PMC3607332

