# Supplementary material for: Relationship between circadian syndrome and stroke: A cross-sectional study of the national health and nutrition examination survey
Source: Front Neurol. 2022 Aug 11;13:946172. doi: 10.3389/fneur.2022.946172 (PMC9403607; doi:10.3389/fneur.2022.946172)
Supplement: Supplementary file 1 [file Table_1.DOCX]

**Supplemental Table 1 Sensitivity analysis of missing data before and after interpolation**

| Variables | Lack of proportion (%) | After the interpolation | Before the interpolation | Statistics | *P* |
| --- | --- | --- | --- | --- | --- |
| Dietary fiber, Mean ± SE | 2.5 | 17.06 ± 0.19 | 17.07 ± 0.19 | t=-0.630 | 0.533 |
| Fat, Mean ± SE | 2.5 | 85.51 ± 0.59 | 85.55 ± 0.60 | t=-0.800 | 0.428 |
| Protein, Mean ± SE | 2.5 | 84.19 ± 0.54 | 84.31 ± 0.55 | t=-1.830 | 0.076 |
| Vitamin A, Mean ± SE | 3.3 | 605.44 ± 7.25 | 604.92 ± 7.32 | t=0.590 | 0.557 |
| Vitamin C, Mean ± SE | 3.6 | 75.93 ± 1.15 | 75.42 ± 1.13 | t=1.110 | 0.102 |
| Vitamin D, Mean ± SE | 2.5 | 4.46 ± 0.07 | 4.46 ± 0.07 | t=-0.280 | 0.781 |
| Vitamin E, Mean ± SE | 2.5 | 8.77 ± 0.10 | 8.78 ± 0.11 | t=-1.730 | 0.086 |
| LDL, Mean ± SE | 1.6 | 113.55 ± 0.47 | 113.91 ± 0.47 | t=-1.600 | 0.090 |
| GHb, Mean ± SE | 0.2 | 5.63 ± 0.01 | 5.63 ± 0.01 | t=-0.120 | 0.903 |
| CRP, Mean ± SE | 0.1 | 1.90 ± 0.06 | 1.90 ± 0.06 | t=1.540 | 0.128 |
| Fruit, Mean ± SE | 2.5 | 0.90 ± 0.02 | 0.90 ± 0.02 | t=-0.810 | 0.418 |
| Vegetable, Mean ± SE | 2.5 | 1.58 ± 0.02 | 1.58 ± 0.02 | t=-1.780 | 0.078 |

**Abbreviations:** LDL: low-density lipoprotein; GHb: glycosylated hemoglubin; CRP: C-reactive protein; Mean ± SE: mean ± standard error.
